# Obesity, Inflammation and Acute Myocardial Infarction - Expression of leptin, IL-6 and high sensitivity-CRP in Chennai based population

**DOI:** 10.1186/1477-9560-10-13

**Published:** 2012-08-14

**Authors:** Karthick Rajendran, Nalini Devarajan, Manohar Ganesan, Malathi Ragunathan

**Affiliations:** 1 Department of Genetics, Dr.ALMPGIBMS, University of Madras, Chennai, -600113, India; 2 Department of Cardiology, Stanley Medical College, Chennai, -600001, India

**Keywords:** Leptin, IL-6, CRP, Obesity, Cardiovascular disease, Acute myocardial infarction

## Abstract

**Background:**

Obesity, characterised by increased fat mass and is currently regarded as a pro-inflammatory state and often associated with increased risk of cardiovascular diseases (CVD) including Myocardial infarction. There is an upregulation of inflammatory markers such as interleukin-6, interleukin-6 receptor and acute phase protein CRP in Acute Myocardial Infarction (AMI) patients but the exact mechanism linking obesity and inflammation is not known. It is of our interest to investigate if serum leptin (*ob* gene product) is associated with AMI and correlated with inflammatory proteins namely Interleukin-6 (IL-6) and high sensitivity - C reactive protein (hs-CRP).

**Results:**

Serum leptin levels were significantly higher in AMI patients when compared to Non-CVD controls. IL-6 and hs-CRP were also elevated in the AMI group and leptin correlated positively with IL-6 and hs-CRP. Incidentally this is the first report from Chennai based population, India.

**Conclusions:**

The strong correlation between serum levels of leptin and IL-6 implicates an involvement of leptin in the upregulation of inflammatory cytokines during AMI. We hypothesise that the increase in values of IL-6, hs-CRP and their correlation to leptin in AMI patients could be due to participation of leptin in the signaling cascade after myocardial ischemia.

## Background

Myocardial Infarction (MI) is a major cause of death globally and worldwide studies have indicated that nine modifiable risk factors including obesity, hypercholesterolemia, hypertension is up to 90% [1] in patients presenting with MI. There are substantial evidences implicating that obesity is a strong risk factor for the development of MI. In addition, even a moderate increase in body mass index (BMI) is associated with an increased risk of heart failure [2]. Patients with Chronic Heart Failure are characterized by metabolic abnormalities the process involving several endocrines including the adipocytic hormone leptin [3].

Leptin, the *ob* gene product was discovered because of its very specific biological action: the ability to regulate body weight and appetite [4]. Leptin is also known to play important role in reproduction, hematopoiesis, immunity and angiogenesis [4,5]. Interestingly, leptin has a structure similar to that of the family of helical cytokines namely interleukins. Thus, leptin shares an extreme functional pleiotropy with other cytokines and is involved in quite diverse physiological functions. Leptin exerts its biological influence by binding to its receptor (OB-R), which belongs to the class I cytokine receptor superfamily [4]. The functional leptin receptors are distributed widely including the hypothalamus, immune cells, myocardium etc.,. In humans, obese individuals possess high values of circulating leptin, which may fail to signal for energy expenditure, suggesting that most human obesity represents a form of leptin resistance.

Reports have suggested that obesity is characterized by inflammation – paralleling the condition with other diseases. It is fairly well established that leptin could be a major link between obesity and inflammation in CVD. A number of inflammation-related proteins are released by adipocytes and these include leptin, cytokines, chemokines and acute phase proteins. Peripheral actions of leptin include endothelial cell activation with subsequent stimulation of pro-inflammatory cytokines [6-8]. Studies have also suggested that leptin is capable of activating immune cells or might initiate a direct action on the vascular wall, thereby leading to initiation and progression of atherosclerosis [9]. In MI patients, who are thrombolysed, severe endothelial dysfunction in the infarct-related arteries is observed [10] with increase in inflammatory cytokines such as IL-6 and also its signaling product CRP. Circulating IL-6 levels constitute a significant pro-atherogenic cytokine and serum IL-6 concentration was an independent predictor of cardiovascular mortality [11]. It is however, not clear as to what extent adipose tissue, particularly leptin contributes quantitatively to the elevated circulating levels of acute phase proteins and cytokines in obesity and whether there is a generalized or local state of inflammation [12].

Previous studies have reported that severity and expression of various factors in CVD depend on different population [13,14]. We therefore focused on Chennai based population with an attempt to understand if serum leptin concentration is associated with myocardial infarction, with particular emphasis on the relationship between serum leptin and inflammatory markers such as IL-6 and CRP in AMI condition. The aim of the present study is 3 fold: ie; 1. to determine circulating leptin, IL-6, hs-CRP levels in both AMI and control subjects and correlate it to BMI, 2. to investigate the relationship between leptin and IL-6, leptin and hs-CRP and 3. to analyse if leptin associated with IL-6 and hs-CRP is an independent predictor of CVD in patients diagnosed for AMI.

## Results

Serum levels of leptin, IL-6 and CRP were evaluated for 93 Acute Myocardial Infarction (AMI) patients and 102 control subjects (Table 1). Their age, BMI, systolic and diastolic blood pressure, pulse rate were recorded. Both Systolic and Diastolic blood pressure and the pulse rate were found to be significantly higher in AMI patients (Table 2).

**Table 1:** Values of BMI, serum leptin, hs-CRP and IL-6 IN AMI and control subjects

**Class**		**N**	**BMI (kg/m**^**2**^**)**	**Leptin (ng/ml)**	**hs-CRP (mg/L)**	**IL-6 (pg/ml)**
Normal weight	Control	21	21.5629±1.32228	15.29±8.616	1.710±0.3434	8.195±2.0532
	AMI	51	20.0673±1.13373	26.70±10.426	5.898±1.7168	22.586±5.450
	P Value		*P<0.030	^**^P<0.007	^**^P<0.001	^**^P<0.001
Over weight	Control	45	24.7096±1.24606	18.85±12.219	2.167±1.3227	11.480±2.7512
	AMI	29	26.0700±1.38817	32.61±14.056	6.721±2.1981	28.128±7.9865
	P Value		*P<0.033	^**^P<0.001	^**^P<0.001	^**^P<0.001
Obese	Control	36	31.6586±3.47939	27.06±12.430	2.450±0.5130	14.408±2.3705
	AMI	13	29.4408±1.4798	42.49±20.277	8.862±1.6322	37.977±6.4761
	P Value	2	**P<0.005	**P<0.002	**P<0.001	**P<0.001

The values are mean±standard deviation. *P=0.05, **P=0.01, is given with the statistical significance between AMI and Control subjects.

**Table 2:** Values of systolic and diastolic blood pressure, pulse rate in AMI and control subjects.

	**Control subjects**	**AMI subjects**	**P Value**
Systolic Blood Pressure	122.21±15.263	138.17±22.027	**P<0.001
Diastolic Blood Pressure	84.26±11.244	94.09±13.612	**P<0.001
Pulse Rate	74.40±7.805	85.19±7.497	**P<0.001

The values are mean±standard deviation. *P=0.05, **P=0.01, is given with the statistical significance between AMI and Control subjects.

Serum concentration of leptin was elevated in AMI Patients compared to controls and this difference was statistically significant. Table 1 shows the increased values of IL-6, hs-CRP in AMI patients and a strong positive correlation was observed between serum levels of leptin and that of IL-6 (r = 0.862, p = 0.001). Correlation was also observed between leptin and hs-CRP (r = 0.318, p = 0.002) in these patients but significant correlation was not observed between leptin and hs-CRP (r = 0.209, p = 0.035), leptin and IL-6 (r = 0.259, p = 0.009) in control subjects (Table 3).

**Table 3:** Spearman’s correlation coefficient analysis of leptin with BMI, hs-CRP and IL-6

**Variables**	**Control subjects**		**AMI subjects**	
	**Leptin**		**Leptin**	
	**R**	**p**	**R**	**p**
BMI	0.337^**^	0.001	0.370^**^	0.001
hs-CRP	0.209^*^	0.035	0.318^**^	0.002
IL-6	0.259^**^	0.009	0.862^**^	0.001

**- Correlation is significant at the 0.01 level.

*- Correlation is significant at the 0.05 level

**Figure 1:**
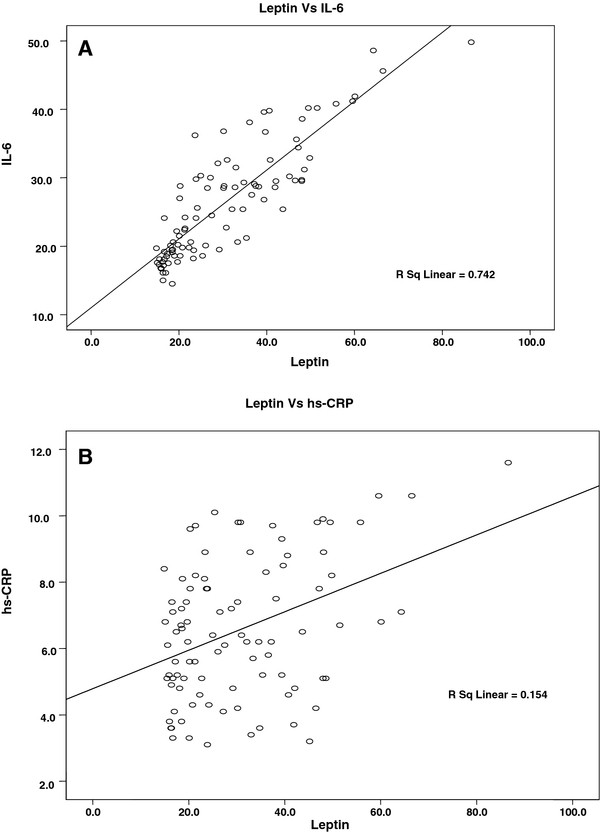
**Correlation between serum levels of leptin (ng/ml), IL-6 (pg/ml) and hs-CRP (mg/L) in patients with AMI (Linear regression analysis).A**. Correlation between serum leptin (ng/ml) and IL-6 (pg/ml) in patients with acute myocardial infarction. Significant positive correlation is found (R^2^ = 0. 742). **B**. Correlation between serum leptin (ng/ml) and hs-CRP (mg/L) in patients with acute myocardial infarction. Positive correlation is found (R^2^ = 0.059).

**Figure 2:**
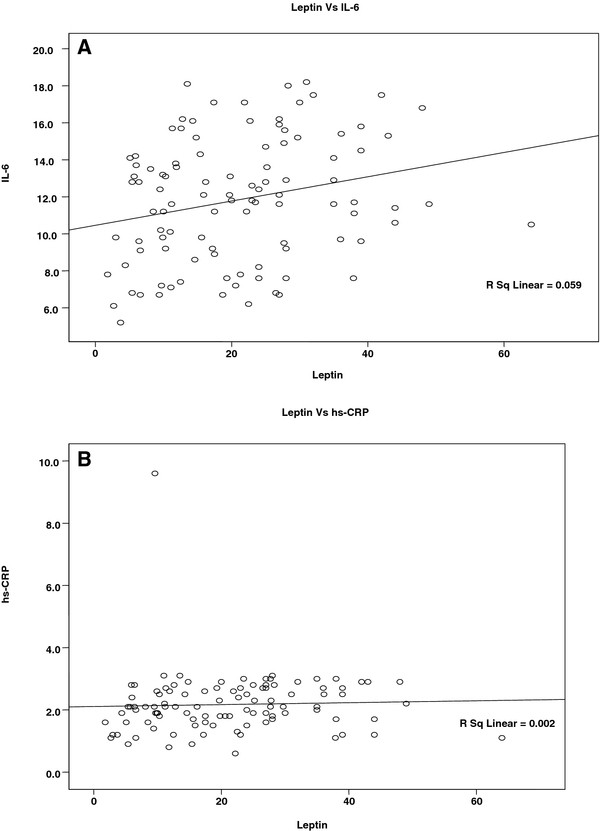
**Correlation between serum levels of leptin (ng/ml), IL-6 (pg/ml) and hs-CRP (mg/L) in control subjects (Linear regression analysis).A**. Correlation between serum leptin (ng/ml) and IL-6 (pg/ml) in control subjects. No significant correlation is found (R^2^ = 0.154). **B**. Correlation between serum leptin (ng/ml) and hs-CRP (mg/L) in control subjects. No significant correlation is found (R^2^ = 0.002).

Correlation between serum parameters such as leptin, IL-6 and hs-CRP was further assessed by linear regression analysis. A positive correlation was found between serum leptin concentration and IL-6 in AMI (R^2^ = 0. 742) (Figure 1A.), while such a relation was not observed in the control group (R^2^ = 0.059) (Figure 2A). A similar analysis has also indicted that serum leptin concentration was related to hs-CRP in AMI (R^2^ = 0.154) (Figure 1B) but not related in control group (R^2^ = 0.002) (Figure 2B).

## Discussion

The objective of our study was to compare values of serum leptin, hs-CRP, IL-6 levels in AMI patients with that of healthy control subjects. Patients with AMI had increased serum levels of leptin compared to control subjects. There was a significant correlation between leptin and BMI in both AMI and control subjects in the three categories including normal, overweight and obese. It is understood that leptin correlates with BMI [15,16], although serum leptin concentration in each AMI group was very high and almost double the values obtained for respective control groups. Interestingly, we found that leptin level of normal weight AMI patients (mean 26.70 ± 10.426 ng/ml) is similar to that of leptin concentration of control obese group (mean 27.06 ± 12.430 ng/ml). This explains that leptin is not only a BMI dependent parameter but in pathological conditions could be determined by inflammation and metabolic defects as observed in AMI patients. Reports have suggested that in humans, serum leptin concentrations reflect the amount of adipose tissue in the body [17]. In obese subjects the observed increase in leptin values is attributed to leptin resistance and hyperleptinemia is associated with elevated blood pressure. In the present study the systolic and diastolic blood pressure and the recorded pulse rate was high in AMI patients.

A strong positive correlation was demonstrated between serum levels of leptin and IL-6 in patients with AMI and control subjects, Serum leptin was found to be correlated with hs-CRP values in both groups. Linear regression analysis was performed further to find the predictor of leptin, markedly higher leptin concentrations correlated with mostly IL-6 (R^2^ = 0.742) than with hs-CRP (R^2^ = 0.154). It appears therefore that leptin concentration is translated into changes in IL-6 concentration possible at the level of IL-6 gene expression. Leptin is not usually thought of as an inflammatory cytokine, hyperleptinemia has been shown to be induced by inflammatory signals [18]. The strong correlation of serum levels of leptin with IL-6 implicates an involvement of leptin in releasing the cytokines during MI. Therefore we suggest that IL-6 secretion is enhanced by leptin in addition to the other factors. Several other studies also suggest that leptin can modulate immune cells by stimulating the production of pro inflammatory cytokines [19-21]. Although we did not evaluate the expression of leptin receptor in our samples, we can speculate that the observed increase in the levels of Leptin, IL-6 and hs-CRP in AMI patients could be due to the presence of an increased number of activated vascular endothelial cells, adipocytes, immune cells, associated with an up regulation of leptin receptor expression.

The findings of the present study are consistent with and compliment the results of previous studies on leptin in chronic heart diseases such as ACS [9] and CHF [22,23]. We have demonstrated for the first time, a relationship between increased levels of leptin, hs-CRP and IL-6 the progressive impairment in Non-Thrombolysed condition of AMI in Chennai based population. Thus, adipocyte-derived hormone leptin might be involved in metabolic abnormalities leading to AMI or leptin might be cardioprotective in ischemia/reperfusion injury [24]. Inflammation and angiogenesis play an important role in healing of injured tissue. In this approach, the activation of the immune system by leptin, together with its angiogenic effect, suggests that leptin may be involved in inflammatory reaction in the infarcted tissue and induce tissue repair.

## Conclusion

High values of leptin as obtained from our study may reflect an ubiquitous susceptibility of peripheral tissues to the action of leptin. The upregulation of IL-6 and the increase in its signaling product hs-CRP suggests the possible involvement of leptin in the signaling cascade through activation of leptin receptor after myocardial ischemia. Our results demonstrate that serum leptin may be a useful marker of inflammation and it may be helpful in assessing the risk of obesity associated CVD. Future studies should focus on the expression of leptin, its receptor in myocardium and their role in underlying pathogenesis of MI.

## Materials and methods

### Population study

The study was carried out on 195 subjects (93 AMI subjects and 102 voluntary BMI, and age matched healthy people as controls) after obtaining written consent. Peripheral venous blood was collected from patients immediately after admission (Non thrombolysed) at ICCU, Department of Cardiology, Stanley Medical Hospital, Chennai. Clinical diagnosis of AMI was made from ECG with ST-segment Elevation (STEMI) and non-ST-segment elevation (Non-STEMI) with typical chest pain persisting for at least 30 min, ST segment elevation > 0.2 mV in at least 2 contiguous leads. From the blood samples collected, serum was isolated after centrifugation at 3,500 rpm and stored at −20°C for subsequent analysis of leptin, hs-CRP and IL-6.

Blood samples were collected from Control group (Non-CVD) at University of Madras Staff quarters, Palavakam, Chennai. Controls comprised of healthy subjects who had no history of chest pain or symptoms suggestive of CVD.

Clinical parameters including blood pressure and pulse rate was measured for all the subjects by trained medical practitioners and anthropometric parameter - BMI was calculated as [weight (kg)/height (m^2^)].

### ELISA assays

Serum leptin was measured using ‘two-step’ sandwich ELISA kit [Diagnostics Biochem Canada Inc. (DBC)]. IL-6 was measured using ‘two-step’ sandwich ELISA kit [Ani Biotech OY, Orgenium Laboratories Business Unit, Finland]. hs-CRP was measured using turbidimetric method [Spinreact, S.AU. Ctra. Santa Coloma]. Laboratory procedures were performed according to manufacturer’s instructions.

### Statistical analysis

Statistical analysis was performed using SPSS software (version 15.0). For better comparison mean ± S.D. is shown. Correlation between variables was examined by estimating Spearman’s correlation coefficient (r_s_) (data not normally distributed). A simple linear regression analysis was performed to identify the predictors of serum leptin. The adjusted coefficient of determination (adjusted R^2^) was used to measure the goodness-of-fit of the final model.

## Abbreviations

CVD, Cardio Vascular Disease; AMI, Acute Myocardial Infarction; hs-CRP, High sensitivity-C Reactive Protein, IL-6, Interleukin 6; BMI, Body Mass Index; BP, Blood pressure.

## Competing interests

The authors declare that they have no competing interests.

## Authors’ contribution

KR carried out the sample collection, experimental studies, statistical analysis and compiled the manuscript, DN helped in collecting the blood samples from non-CVD subjects and standardized leptin ELISA assay, Dr. GM helped in diagnosis of patients and provided the clinical samples, and Dr. MR coordinated and helped in interpreting the data and reviewing the manuscript. All authors read and approved the final manuscript.
